# Association between triglyceride-glucose index and heart failure in patients with type 2 diabetes mellitus: a cross-sectional study

**DOI:** 10.3389/fcvm.2026.1667390

**Published:** 2026-01-27

**Authors:** Tong Yan, Xiaoxia Lv, Jing Chen

**Affiliations:** 1 VIP Health Management Center, Xi'an Gaoxin Hospital, Xi'an, China; 2 Health Medicine Department, Xi'an International Medical Center Hospital, Xi'an, China; 3 Department of Endocrinology, Xi'an Gaoxin Hospital, Xi'an, China

**Keywords:** heart failure, logistic regression, receiver operating characteristic curve, triglyceride-glucose index, type 2 diabetes mellitus

## Abstract

**Objective:**

This study aimed to investigate the association between the triglyceride-glucose (TyG) index and heart failure (HF) in patients with type 2 diabetes mellitus (T2DM).

**Methods:**

This single-center cross-sectional study enrolled 848 hospitalized T2DM patients aged ≥50 years at Xi'an Gaoxin Hospital between January 1, 2024, and January 1, 2025. The TyG index was calculated for each patient, and participants were stratified into three tertiles (T1, T2, T3) based on TyG levels. HF was diagnosed in accordance with the 2021 guidelines of the European Society of Cardiology (ESC). Univariate logistic regression was first used to identify variables significantly associated with HF (*P* < 0.05), which were then included in multivariate logistic regression analyses to examine the independent association between TyG and HF risk. Subgroup analyses were also conducted to examine whether the association between TyG and HF varied by age, sex, smoking status, hypertension, and body mass index (BMI). To evaluate the predictive value of the TyG index for HF, receiver operating characteristic (ROC) curves were plotted, and the area under the curve (AUC) was calculated.

**Results:**

Among the 848 participants, 242 (28.5%) had HF. Higher TyG levels were independently associated with increased HF risk. In the fully adjusted model, the second (T2: OR = 1.655, *P* = 0.026) and third tertiles (T3: OR = 1.762, *P* = 0.018) were significantly associated with elevated odds of HF compared to the lowest tertile (T1). The TyG index as a continuous variable also showed a robust association (OR = 1.493, *P* = 0.002). Subgroup analyses revealed particularly strong associations in individuals aged ≥70 years, males, non-smokers, hypertensive patients, and those with body mass index <24 kg/m^2^. ROC analysis indicated that the TyG index not only showed modest discriminative ability for HF but also enhanced the predictive performance of a baseline model consisting of conventional variables (*P* < 0.05).

**Conclusion:**

Elevated TyG index is independently associated with increased risk of HF in patients with T2DM. It may serve as a clinically accessible, cost-effective biomarker for early HF risk identification, particularly in high-risk subpopulations.

## 1 Introduction

Heart failure (HF) is a common and increasingly burdensome global public health concern, characterized by high morbidity and mortality rates (1). As populations age and metabolic disorders become increasingly prevalent, the burden of HF continues to rise. Among these metabolic disorders, type 2 diabetes mellitus (T2DM) plays a particularly important role, as it has been well established as a major risk factor for HF (2). Epidemiological studies have consistently shown that individuals with T2DM have a significantly higher risk of developing HF than those without diabetes (3–5). The pathophysiological relationship between HF and T2DM is complex and may involve mechanisms such as insulin resistance (IR), chronic low-grade inflammation, oxidative stress, dyslipidemia, and endothelial dysfunction (6). Therefore, early identification of HF risk in patients with T2DM is of critical clinical importance.

In recent years, the triglyceride-glucose (TyG) index has gained attention as a novel and practical surrogate marker for IR (7–9). The TyG index, derived from fasting triglyceride and glucose levels, has been shown to correlate closely with IR and has demonstrated predictive value in various studies (10). It has been associated with coronary artery disease, stroke, atherosclerosis, and cardiovascular mortality, and has also been linked to cardiovascular risk prediction in T2DM populations (11–13). Compared with conventional markers like the Homeostasis Model Assessment of Insulin Resistance (HOMA-IR), the TyG index does not require insulin measurement, making it more accessible in clinical settings, particularly in primary care or resource-limited environments. These advantages make the TyG index a feasible tool for routine risk assessment in real-world clinical practice.

Although the relationship between TyG and various cardiovascular conditions has been explored, evidence regarding its association with HF in patients with T2DM remains limited, especially among high-risk groups. Existing research suggests that elevated TyG levels may be significantly associated with an increased risk of HF (14). However, most existing studies focus on community-based cohorts or general populations, and evidence specifically targeting hospitalized T2DM patients—who usually present with more severe metabolic derangements and higher HF risk—remains scarce. This important gap limits the applicability of prior findings to inpatient clinical settings.

Given this background, the present study aimed to investigate the association between the TyG index and the presence of HF in hospitalized patients with T2DM. By focusing on this high-risk inpatient population, the goal is to provide further clinical evidence supporting the TyG index as a cost-effective and easily obtainable screening tool for HF risk, and to contribute to the development of early identification and personalized management strategies, particularly in high-risk diabetic populations.

## 2 Methods

### 2.1 Study design and population

This was a single-center, cross-sectional study. Participants were hospitalized patients with T2DM admitted to Xi'an Gaoxin Hospital between January 1, 2024, and January 1, 2025. Inclusion criteria were: (1) age ≥ 50 years, a threshold selected because older adults with T2DM are more likely to exhibit metabolic abnormalities and early manifestations of HF, making this group clinically relevant for HF risk assessment; (2) diagnosis of T2DM based on the criteria established by the American Diabetes Association (ADA) (15); (3) availability of complete echocardiographic data. Exclusion criteria included: (1) type 1 diabetes or other specific types of diabetes (such as gestational diabetes, monogenic diabetes); (2) concurrent malignancy, acute or chronic infections, or severe hepatic or renal dysfunction; (3) known structural heart diseases such as rheumatic valvular disease, congenital heart disease, or dilated cardiomyopathy; (4) serious hematological disorders (such as leukemia or lymphoma); (5) missing data on fasting triglycerides or FBG; (6) acute HF at the time of admission. This study was reviewed and approved by the Ethics Committee of Xi'an Gaoxin Hospital. Written informed consent was obtained from all participants. The study procedures complied with the ethical principles outlined in the Declaration of Helsinki.

### 2.2 Definition and classification of TyG index

The TyG index was calculated using the formula: TyG = ln [fasting triglycerides (mg/dL) × FBG (mg/dL)/2] (16). To ensure consistency, triglyceride and FBG values originally measured in mmol/L were converted to mg/dL by multiplying by 88.5 and 18, respectively. Based on TyG tertiles, participants were classified into three groups: the first tertile (T1) included individuals with TyG ≤ 9.03 (*n* = 283), the second tertile (T2) included those with 9.03 < TyG ≤ 9.62 (*n* = 283), and the third tertile (T3) included those with TyG > 9.62 (*n* = 282).

### 2.3 Definition and classification of heart failure

HF was identified using clinical and echocardiographic data, following the recommendations of the 2021 guidelines from the European Society of Cardiology (17). Based on the presence or absence of HF, participants were categorized into an HF group (*n* = 242) and a non-HF group (*n* = 606) for comparative analysis of clinical features and TyG index levels.

### 2.4 Assessment and definition of covariates

A range of covariates potentially influencing the relationship between the TyG index and HF were collected in this study, including demographic characteristics, clinical measurements, laboratory indicators, and medication history. Demographic data consisted of age, sex, and smoking status. Smoking was defined as having smoked at least one cigarette per day for a duration of six months or more, either currently or in the past. Hypertension was identified based on a prior clinical diagnosis, current use of antihypertensive medications, or measured blood pressure with systolic ≥140 mmHg and/or diastolic ≥90 mmHg (18). T2DM was diagnosed based on the criteria of the ADA, which include one or more of the following: a FBG level ≥7.0 mmol/L, 2 h plasma glucose ≥11.1 mmol/L during a 75 g oral glucose tolerance test (OGTT), glycated hemoglobin (HbA1c) ≥ 6.5%, or a documented history of T2DM with ongoing treatment (15). Physical measurements were performed by trained nurses and included height (cm), weight (kg), and body mass index (BMI), calculated as weight divided by height squared (kg/m^2^). Resting heart rate (beats per minute) was recorded at admission, typically using electrocardiogram monitoring. Laboratory parameters were assessed from fasting venous blood samples collected in the early morning. The following assays were used: FBG measured via glucose oxidase method (mmol/L), HbA1c by high-performance liquid chromatography (%), and lipid profiles including triglycerides, total cholesterol, low density lipoprotein cholesterol (LDL-C), and high density lipoprotein cholesterol (HDL-C) using enzymatic colorimetric methods (mmol/L). Hematologic indices including white blood cell count (WBC), hemoglobin, and platelet count were determined using automated hematology analyzers, reported in ×10^9^/L and g/L as appropriate. Liver function tests included alanine aminotransferase (ALT) and aspartate aminotransferase (AST) via rate method (U/L), total bilirubin by dichlorophenol sulfonate colorimetric assay (μmol/L), and albumin via bromocresol green method (g/L). Renal function was evaluated using serum creatinine (enzymatic method, μmol/L), blood urea nitrogen (BUN; urease-UV method, mmol/L), and uric acid (uricase method, μmol/L). Fibrinogen was measured using the Clauss method (g/L). Electrolytes including sodium, potassium, and calcium were measured by ion-selective electrode technique (mmol/L). Medication use was recorded based on electronic medical records or physician documentation, specifically noting current use of antihypertensive or antidiabetic drugs. All data were collected by trained clinical staff and reviewed independently by two researchers to ensure completeness and accuracy.

### 2.5 Statistical methods

All statistical analyses were performed using IBM SPSS Statistics for Windows, Version 26.0 (IBM Corp., Armonk, NY, USA), and R software (version 4.4.3; R Foundation for Statistical Computing, Vienna, Austria). Continuous variables were expressed as medians with interquartile ranges. The Mann–Whitney *U* test was used for comparisons between two groups, while the Kruskal–Wallis H test was applied for comparisons among multiple groups. Categorical variables were presented as frequencies (percentages), and differences between groups were assessed using the Pearson chi-square test. To explore the association between the TyG index and HF in patients T2DM, univariate logistic regression analyses were first conducted to identify variables significantly associated with HF (*P* < 0.05). These variables were then included in multivariate logistic regression models, constructed in three steps: Model 1 adjusted for age only; Model 2 additionally adjusted for smoking status, hypertension, and heart rate; Model 3 further adjusted for WBC, hemoglobin, HbA1c, HDL-C, uric acid, BUN, creatinine, AST, albumin, fibrinogen, serum sodium, calcium, and potassium. Odds ratios (ORs) and 95% confidence intervals (CIs) were reported for all regression results. To evaluate the predictive value of the TyG index for HF, receiver operating characteristic (ROC) curves were plotted, and the area under the curve (AUC) with corresponding 95% CIs was calculated. In addition to the TyG index alone, we constructed a baseline model using conventional clinical variables (including creatinine), and further built a combined model incorporating TyG (baseline + TyG). ROC curves were compared using DeLong's test to assess whether the difference in AUCs was statistically significant. Moreover, to assess the incremental predictive ability of TyG, net reclassification improvement (NRI) and integrated discrimination improvement (IDI) were calculated. Subgroup analyses were also conducted to examine whether the association between TyG and HF varied by age, sex, smoking status, hypertension, and BMI. All statistical tests were two-sided, and a *P*-value of less than 0.05 was considered statistically significant.

## 3 Results

### 3.1 Baseline characteristics according to TyG tertiles

As shown in Table 1, multiple baseline characteristics demonstrated significant differences across TyG tertiles. Compared with the lowest TyG tertile (T1), individuals in the highest tertile (T3) were younger (*P* < 0.001), had a higher proportion of smokers (*P* = 0.017), and showed significantly lower prevalence of hypertension (*P* < 0.001) and use of antihypertensive medications (*P* < 0.001). The proportion of individuals using antidiabetic drugs also declined with increasing TyG levels (*P* = 0.002). Additionally, heart rate (*P* = 0.010), WBC (*P* = 0.038), hemoglobin (*P* = 0.025), FBG (*P* < 0.001), HbA1c (*P* < 0.001), triglycerides (*P* < 0.001), total cholesterol (*P* < 0.001), and LDL-C (*P* < 0.001) all increased, whereas HDL-C decreased (*P* < 0.001) with rising TyG tertiles. Levels of uric acid (*P* < 0.001), ALT (*P* < 0.001), AST (*P* = 0.013), and fibrinogen (*P* = 0.003) also rose with higher TyG. Differences were also observed in serum sodium (*P* < 0.001) and calcium (*P* = 0.002), with total bilirubin being lowest in the T3 group (*P* < 0.001). Finally, the incidence of HF was highest in T3 and lowest in T1, with a statistically significant trend (*P* < 0.001).

**Table 1:** Baseline characteristics according to TyG tertiles.

Variables	Overall	T1	T2	T3	*P* value
*N*	848	283	283	282	
Age, years	67.00 (61.00, 73.50)	68.00 (63.00, 76.00)	67.00 (60.00, 73.00)	66.00 (59.00, 71.00)	<0.001
Sex, *n* (%)					0.189
Male	433 (51.1%)	157 (55.5%)	139 (49.1%)	137 (48.6%)	
Female	415 (48.9%)	126 (44.5%)	144 (50.9%)	145 (51.4%)	
Smoking, *n* (%)	245 (28.9%)	65 (23.0%)	85 (30.0%)	95 (33.7%)	0.017
Hypertension, *n* (%)	534 (63.0%)	218 (77.0%)	169 (59.7%)	147 (52.1%)	<0.001
Antihypertensive drugs, *n* (%)	477 (56.3%)	201 (71.0%)	166 (58.7%)	110 (39.0%)	<0.001
Antidiabetic drugs, *n* (%)	682 (80.4%)	247 (87.3%)	218 (77.0%)	217 (77.0%)	0.002
BMI, kg/m^2^	25.25 (23.44, 27.13)	25.21 (23.44, 27.06)	25.25 (23.36, 27.43)	25.35 (23.63, 27.06)	0.821
SBP, mmHg	148.00 (133.00, 164.00)	148.00 (132.00, 164.00)	147.00 (132.00, 162.00)	149.00 (135.00, 166.00)	0.353
DBP, mmHg	85.00 (77.00, 94.00)	85.00 (78.00, 93.00)	84.00 (76.00, 95.00)	86.00 (78.00, 94.00)	0.416
Heart rate, times/minute	82.0 (71.0, 95.5)	80.0 (70.0, 90.0)	83.0 (72.0, 96.0)	84.0 (74.0, 96.0)	0.010
WBC, ×10^9^/L	6.9 (5.8, 8.5)	6.8 (5.6, 8.1)	6.9 (5.8, 8.6)	7.1 (6.0, 8.8)	0.038
Hemoglobin, g/L	137.00 (126.00, 149.00)	136.00 (124.00, 147.00)	138.00 (126.00, 150.00)	139.00 (129.00, 151.00)	0.025
Platelet count, ×10^9^/L	214.00 (180.00, 254.00)	211.00 (178.00, 245.00)	221.00 (180.00, 257.00)	215.00 (182.00, 257.00)	0.142
Fasting blood glucose, mmol/L	8.46 (7.03, 11.31)	7.06 (5.70, 8.01)	8.79 (7.27, 10.65)	11.63 (8.77, 14.33)	<0.001
Glycated hemoglobin, %	7.40 (6.60, 8.10)	7.00 (6.30, 7.50)	7.30 (6.50, 8.10)	7.80 (7.10, 8.80)	<0.001
Triglycerides, mmol/L	1.59 (1.11, 2.35)	0.97 (0.77, 1.23)	1.62 (1.30, 1.89)	2.90 (2.09, 3.94)	<0.001
Total cholesterol, mmol/L	4.43 (3.65, 5.22)	4.03 (3.24, 4.93)	4.41 (3.67, 5.14)	4.90 (4.21, 5.73)	<0.001
LDL-C, mmol/L	2.73 (2.08, 3.45)	2.52 (1.80, 3.13)	2.72 (2.14, 3.48)	2.94 (2.37, 3.71)	<0.001
HDL-C, mmol/L	1.11 (0.94, 1.31)	1.16 (0.99, 1.36)	1.10 (0.94, 1.30)	1.07 (0.90, 1.26)	<0.001
Uric acid, μmol/L	316.00 (251.00, 382.00)	299.00 (246.00, 359.00)	315.00 (251.00, 392.00)	337.00 (269.00, 398.00)	<0.001
Blood urea nitrogen, mmol/L	5.77 (4.71, 7.17)	6.00 (4.87, 7.25)	5.68 (4.60, 7.15)	5.78 (4.78, 7.07)	0.474
Creatinine, μmol/L	64.00 (53.00, 79.00)	65.00 (53.00, 78.00)	65.00 (54.00, 80.00)	63.50 (52.00, 79.00)	0.513
ALT, U/L	20.00 (15.00, 31.00)	19.00 (14.00, 26.00)	21.00 (15.00, 33.00)	22.00 (16.00, 35.00)	<0.001
AST, U/L	21.00 (16.00, 28.00)	19.00 (16.00, 24.00)	21.00 (16.00, 28.00)	22.00 (17.00, 33.00)	0.013
Total bilirubin, μmol/L	12.20 (9.30, 16.20)	13.57 (10.00, 17.60)	12.20 (9.40, 15.70)	11.30 (8.40, 15.50)	<0.001
Albumin, g/L	42.00 (39.00, 45.00)	42.00 (39.00, 45.00)	42.00 (39.00, 45.00)	42.00 (39.00, 45.00)	0.948
Fibrinogen, g/L	3.30 (2.81, 3.89)	3.25 (2.77, 3.88)	3.47 (2.92, 4.12)	3.20 (2.77, 3.79)	0.003
Serum sodium, mmol/L	140.00 (138.00, 142.00)	141.00 (139.00, 143.00)	140.00 (138.00, 143.00)	139.50 (137.00, 142.00)	<0.001
Serum calcium, mmol/L	2.24 (2.17, 2.30)	2.24 (2.14, 2.28)	2.24 (2.16, 2.30)	2.24 (2.21, 2.33)	0.002
Serum potassium, mmol/L	4.12 (3.85, 4.38)	4.12 (3.87, 4.35)	4.13 (3.85, 4.41)	4.12 (3.79, 4.39)	0.543
LVEF, %	62.00 (59.00, 65.00)	62.00 (59.00, 65.00)	62.00 (58.00, 64.00)	61.00 (59.00, 65.00)	0.458
Heart failure, *n* (%)					<0.001
No	606 (71.5%)	225 (79.5%)	196 (69.3%)	185 (65.6%)	
Yes	242 (28.5%)	58 (20.5%)	87 (30.7%)	97 (34.4%)	

T1: TyG ≤ 9.03; T2: 9.03 < TyG ≤ 9.62; T3: TyG > 9.62; TyG, triglyceride-glucose index; BMI, body mass index; SBP, systolic blood pressure; DBP, diastolic blood pressure; WBC, white blood cell count; LDL-C, low-density lipoprotein cholesterol; HDL-C, high-density lipoprotein cholesterol; ALT, alanine aminotransferase; AST, aspartate aminotransferase; LVEF, left ventricular ejection fraction.

### 3.2 Baseline characteristics based on heart failure classification

As shown in Table 2, patients with HF exhibited significant differences in several baseline characteristics compared to those without HF. Individuals in the HF group were older (*P* < 0.001), had a higher proportion of smokers (*P* = 0.018), and a higher prevalence of hypertension (*P* = 0.006). Heart rate was significantly elevated in the HF group (*P* = 0.027), along with increased WBC (*P* < 0.001), while hemoglobin levels were lower (*P* = 0.038). Metabolic indicators showed that FBG (*P* < 0.001) and HbA1c (*P* < 0.001) were significantly higher in the HF group, while HDL-C was lower (*P* = 0.003). Additionally, levels of uric acid (*P* < 0.001), BUN (*P* < 0.001), creatinine (*P* < 0.001), and AST (*P* < 0.001) were all elevated in the HF group. In terms of nutritional and inflammatory markers, serum albumin was significantly decreased (*P* < 0.001), whereas fibrinogen was increased (*P* < 0.001). Electrolyte analysis revealed that serum sodium (*P* = 0.002), calcium (*P* < 0.001), and potassium (*P* = 0.027) were all lower in HF patients. Moreover, the TyG index was significantly higher in the HF group (*P* < 0.001), while LVEF was markedly reduced (*P* < 0.001).

**Table 2:** Baseline characteristics based on heart failure classification.

Variables	Non-HF	HF	*P* value
*N*	606	242	
Age, years	66.00 (60.00, 72.00)	70.00 (63.00, 78.00)	<0.001
Sex, *n* (%)			0.082
Male	298 (49.2%)	135 (55.8%)	
Female	308 (50.8%)	107 (44.2%)	
Smoking, *n* (%)	161 (26.6%)	84 (34.7%)	0.018
Hypertension, *n* (%)	364 (60.1%)	170 (70.2%)	0.006
Antihypertensive drugs, *n* (%)	345 (56.9%)	132 (54.5%)	0.527
Antidiabetic drugs, *n* (%)	494 (81.5%)	188 (77.7%)	0.204
BMI, kg/m^2^	25.25 (23.44, 27.24)	25.25 (23.34, 27.04)	0.503
SBP, mmHg	149.00 (135.00, 164.00)	146.00 (131.00, 164.00)	0.111
DBP, mmHg	85.00 (78.00, 93.00)	86.00 (77.00, 97.00)	0.346
Heart rate, times/minute	82.00 (70.00, 94.00)	82.00 (72.00, 98.00)	0.027
WBC, ×10^9^/L	6.80 (5.70, 8.10)	7.50 (6.03, 9.45)	<0.001
Hemoglobin, g/L	138.00 (128.00, 149.00)	135.50 (121.00, 148.00)	0.038
Platelet count, ×10^9^/L	213.00 (181.00, 255.00)	215.00 (177.00, 252.00)	0.779
Fasting blood glucose, mmol/L	7.94 (6.66, 10.38)	10.19 (8.06, 12.78)	<0.001
Glycated hemoglobin, %	7.30 (6.50, 7.90)	7.50 (6.80, 8.60)	<0.001
Triglycerides, mmol/L	1.57 (1.04, 2.36)	1.61 (1.22, 2.33)	0.172
Total cholesterol, mmol/L	4.47 (3.61, 5.27)	4.41 (3.72, 5.13)	0.482
LDL-C, mmol/L	2.73 (2.06, 3.46)	2.73 (2.10, 3.45)	0.724
HDL-C, mmol/L	1.12 (0.96, 1.32)	1.10 (0.88, 1.24)	0.003
Uric acid, μmol/L	309.50 (248.00, 371.00)	342.50 (274.00, 413.00)	<0.001
Blood urea nitrogen, mmol/L	5.66 (4.66, 6.89)	6.24 (4.87, 8.30)	<0.001
Creatinine, μmol/L	61.00 (52.00, 74.00)	73.00 (58.00, 94.00)	<0.001
ALT, U/L	20.00 (15.00, 30.00)	21.00 (15.00, 35.00)	0.099
AST, U/L	20.00 (16.00, 25.00)	22.00 (17.00, 36.00)	<0.001
Total bilirubin, μmol/L	12.40 (9.50, 15.80)	12.10 (8.60, 17.10)	0.977
Albumin, g/L	43.00 (40.00, 45.00)	40.00 (37.00, 43.00)	<0.001
Fibrinogen, g/L	3.23 (2.77, 3.78)	3.51 (3.00, 4.28)	<0.001
Serum sodium, mmol/L	140.00 (139.00, 143.00)	140.00 (137.00, 142.00)	0.002
Serum calcium, mmol/L	2.24 (2.19, 2.31)	2.24 (2.12, 2.27)	<0.001
Serum potassium, mmol/L	4.13 (3.89, 4.38)	4.08 (3.76, 4.36)	0.027
TyG	9.26 (8.75, 9.77)	9.48 (9.05, 9.91)	<0.001
LVEF, %	63.00 (60.00, 65.00)	57.00 (47.00, 61.00)	<0.001

HF, heart failure; BMI, body mass index; SBP, systolic blood pressure; DBP, diastolic blood pressure; WBC, white blood cell count; LDL-C, low-density lipoprotein cholesterol; HDL-C, high-density lipoprotein cholesterol; ALT, alanine aminotransferase; AST, aspartate aminotransferase; LVEF, left ventricular ejection fraction; TyG, triglyceride-glucose index.

### 3.3 Univariate and multivariate logistic regression analysis of heart failure

As shown in Table 3, univariate logistic regression analysis revealed several variables significantly associated with HF. Increasing age was linked to a higher risk of HF (OR = 1.046, *P* < 0.001), as was smoking (OR = 1.469, *P* = 0.018) and hypertension (OR = 1.570, *P* = 0.006). Elevated heart rate (OR = 1.015, *P* < 0.001), WBC (OR = 1.201, *P* < 0.001), FBG (OR = 1.134, *P* < 0.001), HbA1c (OR = 1.254, *P* < 0.001), uric acid (OR = 1.004, *P* < 0.001), BUN (OR = 1.114, *P* < 0.001), creatinine (OR = 1.016, *P* < 0.001), AST (OR = 1.004, *P* = 0.004), and fibrinogen (OR = 1.332, *P* < 0.001) were all positively associated with HF. Conversely, hemoglobin (OR = 0.989, *P* = 0.009), HDL-C (OR = 0.516, *P* = 0.013), albumin (OR = 0.863, *P* < 0.001), serum sodium (OR = 0.950, *P* = 0.005), serum calcium (OR = 0.042, *P* < 0.001), and serum potassium (OR = 0.630, *P* = 0.012) were inversely associated with HF, suggesting protective effects. Importantly, the TyG index showed a strong positive association with HF risk (OR = 1.549, *P* < 0.001). Compared to the lowest TyG tertile (T1), individuals in the second (T2: OR = 1.722, *P* = 0.005) and third tertiles (T3: OR = 2.034, *P* < 0.001) had significantly higher odds of developing HF.

**Table 3:** Univariate and multivariate logistic regression analysis of heart failure.

Variables	OR	95% CI	*P* value	OR	95% CI	*P* value
Age	1.046	1.028, 1.063	<0.001	1.047	1.025, 1.069	<0.001
Sex						
Male	Reference					
Female	0.767	0.568, 1.035	0.082			
Smoking	1.469	1.067, 2.024	0.018	1.805	1.232, 2.645	0.002
Hypertension	1.570	1.140, 2.162	0.006	1.658	1.149, 2.393	0.007
Antihypertensive drugs	0.908	0.673, 1.225	0.527			
Antidiabetic drugs	0.789	0.548, 1.138	0.205			
BMI	0.982	0.935, 1.031	0.466			
SBP	0.995	0.989, 1.001	0.112			
DBP	1.005	0.994, 1.016	0.340			
Heart rate	1.015	1.007, 1.023	<0.001	1.010	1.001, 1.020	0.027
WBC	1.201	1.125, 1.281	<0.001	1.088	1.006, 1.176	0.034
Hemoglobin	0.989	0.981, 0.997	0.009	0.999	0.988, 1.010	0.858
Platelet count	0.999	0.997, 1.002	0.657			
Fasting blood glucose	1.134	1.090, 1.181	<0.001			
Glycated hemoglobin	1.254	1.124, 1.399	<0.001	1.215	1.054, 1.401	0.007
Triglycerides	0.976	0.885, 1.077	0.628			
Total cholesterol	0.958	0.846, 1.085	0.502			
LDL-C	1.014	0.871, 1.180	0.857			
HDL-C	0.516	0.306, 0.869	0.013	0.913	0.532, 1.567	0.742
Uric acid	1.004	1.002, 1.005	<0.001	1.003	1.001, 1.004	0.006
Blood urea nitrogen	1.114	1.048, 1.183	<0.001	1.005	0.941, 1.074	0.877
Creatinine	1.016	1.010, 1.022	<0.001	1.009	1.003, 1.015	0.002
ALT	1.001	0.999, 1.003	0.273			
AST	1.004	1.001, 1.007	0.004	1.003	1.000, 1.006	0.039
Total bilirubin	1.005	0.983, 1.028	0.637			
Albumin	0.863	0.831, 0.896	<0.001	0.917	0.878, 0.956	<0.001
Fibrinogen	1.332	1.165, 1.523	<0.001	1.100	0.953, 1.270	0.191
Serum sodium	0.950	0.916, 0.985	0.005	0.999	0.959, 1.040	0.943
Serum calcium	0.042	0.012, 0.145	<0.001	0.228	0.043, 1.208	0.082
Serum potassium	0.630	0.440, 0.903	0.012	0.512	0.338, 0.775	0.002
TyG	1.549	1.270, 1.891	<0.001	1.493	1.156–1.929	0.002
TyG						
T1	Reference			Reference		
T2	1.722	1.173, 2.527	0.005	1.655	1.063–2.578	0.026
T3	2.034	1.392, 2.971	<0.001	1.762	1.103–2.814	0.018

T1: TyG ≤ 9.03; T2: 9.03 < TyG ≤ 9.62; T3: TyG > 9.62; TyG, triglyceride-glucose index; BMI, body mass index; SBP, systolic blood pressure; DBP, diastolic blood pressure; WBC, white blood cell count; LDL-C, low-density lipoprotein cholesterol; HDL-C, high-density lipoprotein cholesterol; ALT, alanine aminotransferase; AST, aspartate aminotransferase.

In multivariate logistic regression analysis (right panel of Table 3), several variables remained significantly associated with HF after adjusting for confounders. Age (OR = 1.047, *P* < 0.001), smoking (OR = 1.805, *P* = 0.002), hypertension (OR = 1.658, *P* = 0.007), heart rate (OR = 1.010, *P* = 0.027), WBC (OR = 1.088, *P* = 0.034), HbA1c (OR = 1.215, *P* = 0.007), uric acid (OR = 1.003, *P* = 0.006), creatinine (OR = 1.009, *P* = 0.002), AST (OR = 1.003, *P* = 0.039), albumin (OR = 0.917, *P* < 0.001), and serum potassium (OR = 0.512, *P* = 0.002) remained independent predictors. The TyG index remained an independent risk factor for HF (OR = 1.493, *P* = 0.002). Moreover, compared to T1, both T2 (OR = 1.655, *P* = 0.026) and T3 (OR = 1.762, *P* = 0.018) were independently associated with increased HF risk.

### 3.4 Multivariate logistic regression analysis of TyG and heart failure

As shown in Table 4, multivariate logistic regression analysis demonstrated that higher TyG levels were consistently associated with an increased risk of HF. In Model 1, adjusted for age only, both the middle (T2: OR = 1.975, *P* = 0.001) and highest TyG tertiles (T3: OR = 2.508, *P* < 0.001) showed significantly higher odds of HF compared to the lowest tertile (T1), and TyG as a continuous variable was also strongly associated (OR = 1.765, *P* < 0.001). After further adjusting for smoking, hypertension, and heart rate in Model 2, the associations remained significant (T2: OR = 2.050, *P* = 0.001; T3: OR = 2.694, *P* < 0.001; TyG: OR = 1.809, *P* < 0.001). Even in Model 3, which included a comprehensive set of clinical covariates, both T2 (OR = 1.655, *P* = 0.026) and T3 (OR = 1.762, *P* = 0.018) remained significantly associated with HF, as did the TyG index as a continuous variable (OR = 1.493, *P* = 0.002).

**Table 4:** Multivariate logistic regression analysis of TyG and heart failure.

Variables	Model 1			Model 2			Model 3		
	OR	95% CI	*P* value	OR	95% CI	*P* value	OR	95% CI	*P* value
T1	Reference			Reference			Reference		
T2	1.975	1.330–2.933	0.001	2.050	1.364–3.082	0.001	1.655	1.063–2.578	0.026
T3	2.508	1.689–3.726	<0.001	2.694	1.780–4.078	<0.001	1.762	1.103–2.814	0.018
TyG	1.765	1.433–2.173	<0.001	1.809	1.455–2.250	<0.001	1.493	1.156–1.929	0.002

T1: TyG ≤ 9.03; T2: 9.03 < TyG ≤ 9.62; T3: TyG > 9.62; Model 1: Adjusted for age only; Model 2: Adjusted for age, smoking, hypertension, and heart rate; Model 3: Adjusted for age, smoking, hypertension, heart rate, white blood cell count, hemoglobin, glycated hemoglobin, high-density lipoprotein cholesterol, uric acid, blood urea nitrogen, creatinine, aspartate aminotransferase, albumin, fibrinogen, sodium, potassium, and calcium. TyG, triglyceride-glucose index; OR, odds ratio; CI, confidence interval.

### 3.5 Subgroup analysis of TyG and heart failure

As shown in Table 5, subgroup analysis revealed significant associations between higher TyG levels and HF in specific populations. Among individuals aged ≥ 70 years, both T2 and T3 groups had markedly higher risks of HF compared to T1 (T2: OR = 2.819, *P* = 0.001; T3: OR = 4.385, *P* < 0.001), and TyG as a continuous variable was also strongly associated (OR = 3.049, *P* < 0.001). In males, the T2 group showed increased risk (OR = 2.035, *P* = 0.017), and TyG remained significant (OR = 1.437, *P* = 0.022). Among non-smokers, T2 was significantly associated with higher HF risk (OR = 1.977, *P* = 0.011), and TyG was also significant (OR = 1.557, *P* = 0.006). For individuals with hypertension, both T2 and T3 groups had elevated risks (T2: OR = 2.556, *P* = 0.001; T3: OR = 3.795, *P* < 0.001), and TyG was again significantly associated (OR = 2.723, *P* < 0.001). Interestingly, in those without hypertension, the T3 group showed a decreased risk (OR = 0.388, *P* = 0.036), and TyG was negatively associated with HF (OR = 0.527, *P* = 0.022). In participants with BMI < 24 kg/m^2^, T2 and T3 were both significantly associated with increased HF risk (T2: OR = 3.022, *P* = 0.006; T3: OR = 4.212, *P* < 0.001), with TyG also significant (OR = 1.797, *P* = 0.002). Among those with BMI ≥ 24 kg/m^2^, while T2 and T3 were not statistically significant, TyG remained positively associated (OR = 1.542, *P* = 0.009). Overall, these subgroup findings should be interpreted cautiously, as limited sample sizes in certain categories may affect the stability and generalizability of the estimates.

**Table 5:** Subgroup analysis of TyG and heart failure.

Subgroups	T2 vs. T1			TyG			TyG		
	OR	95% CI	*P* value	OR	95% CI	*P* value	OR	95% CI	*P* value
Age									
< 70 years	0.871	0.444–1.708	0.688	1.007	0.507–1.999	0.985	0.983	0.673–1.435	0.928
≥ 70 years	2.819	1.541–5.157	0.001	4.385	2.308–8.334	<0.001	3.049	2.060–4.514	<0.001
Sex									
Male	2.035	1.138–3.640	0.017	1.714	0.953–3.083	0.072	1.437	1.053–1.963	0.022
Female	1.092	0.526–2.268	0.813	1.215	0.565–2.613	0.618	1.288	0.836–1.982	0.251
Smoking									
Yes	0.770	0.318–1.866	0.563	1.506	0.621–3.654	0.365	1.283	0.798–2.064	0.304
No	1.977	1.170–3.341	0.011	1.594	0.890–2.858	0.117	1.557	1.132–2.142	0.006
Hypertension									
Yes	2.556	1.478–4.421	0.001	3.795	2.124–6.782	<0.001	2.723	2.016–3.677	<0.001
No	0.574	0.245–1.344	0.201	0.388	0.160–0.939	0.036	0.527	0.304–0.913	0.022
BMI									
<24 kg/m^2^	3.022	1.371–6.661	0.006	4.212	1.915–9.266	<0.001	1.797	1.229–2.628	0.002
≥24 Kg/m^2^	1.350	0.779–2.338	0.285	1.300	0.733–2.305	0.369	1.542	1.116–2.131	0.009

T1: TyG ≤ 9.03; T2: 9.03 < TyG ≤ 9.62; T3: TyG > 9.62; TyG, triglyceride-glucose index; BMI, body mass index; OR, odds ratio; CI, confidence interval.

### 3.6 ROC curve evaluating the predictive value of TyG for heart failure

**Figure 1:**
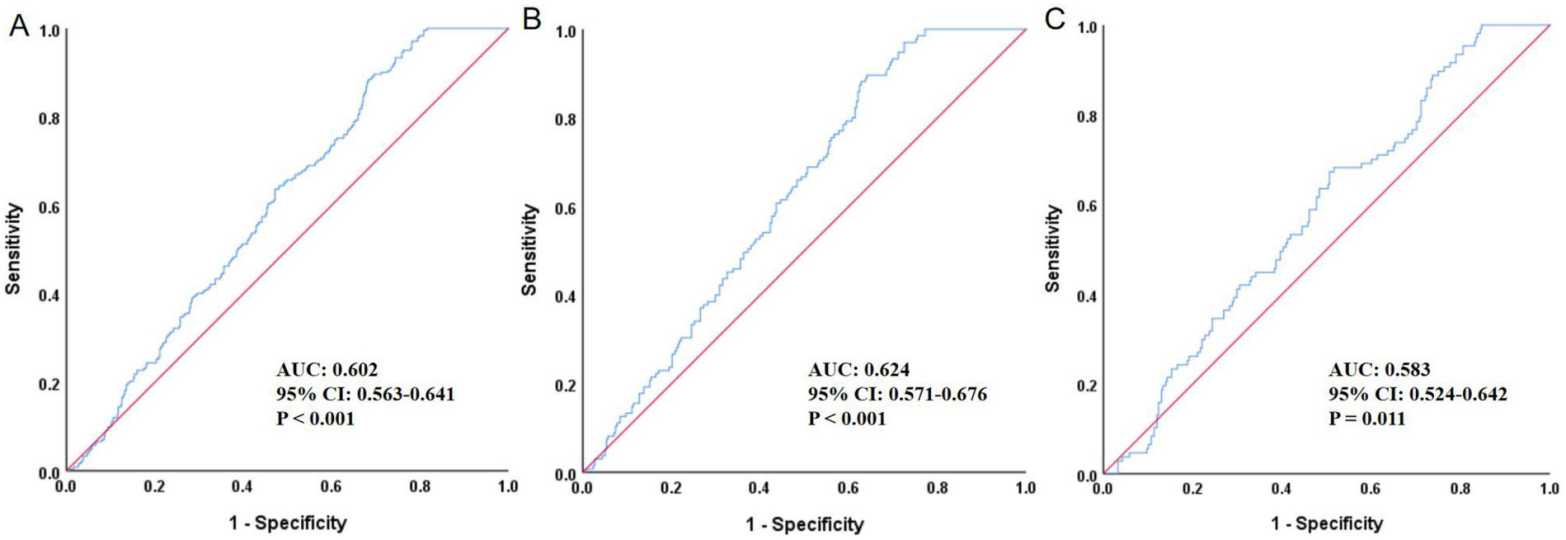
ROC curve evaluating the predictive value of TyG for heart failure. **(A)** Total population, **(B)** Male, **(C)** Female; ROC, receiver operating characteristic; TyG, triglyceride-glucose index; AUC, area under the curve; CI, confidence interval.

Figure 1 presented the ROC curve analysis of the TyG index for predicting HF. In the total population (Panel A), the AUC was 0.602 (95% CI: 0.563–0.641, *P* < 0.001), indicating statistical significance but only modest discriminatory ability. The optimal cut-off value for the total population was 8.88. Among males (Panel B), the AUC was 0.624 (95% CI: 0.571–0.676, *P* < 0.001), showing slightly better—yet still modest—predictive performance. The optimal cut-off in males was 8.86. In females (Panel C), the AUC was 0.583 (95% CI: 0.524–0.642, *P* = 0.011), which was lower but still statistically significant. The optimal cut-off in females was 9.29; however, these results should be interpreted cautiously given the relatively smaller subgroup size. Overall, the TyG index demonstrated a modest predictive value for HF, with slightly stronger performance observed in males.

**Figure 2:**
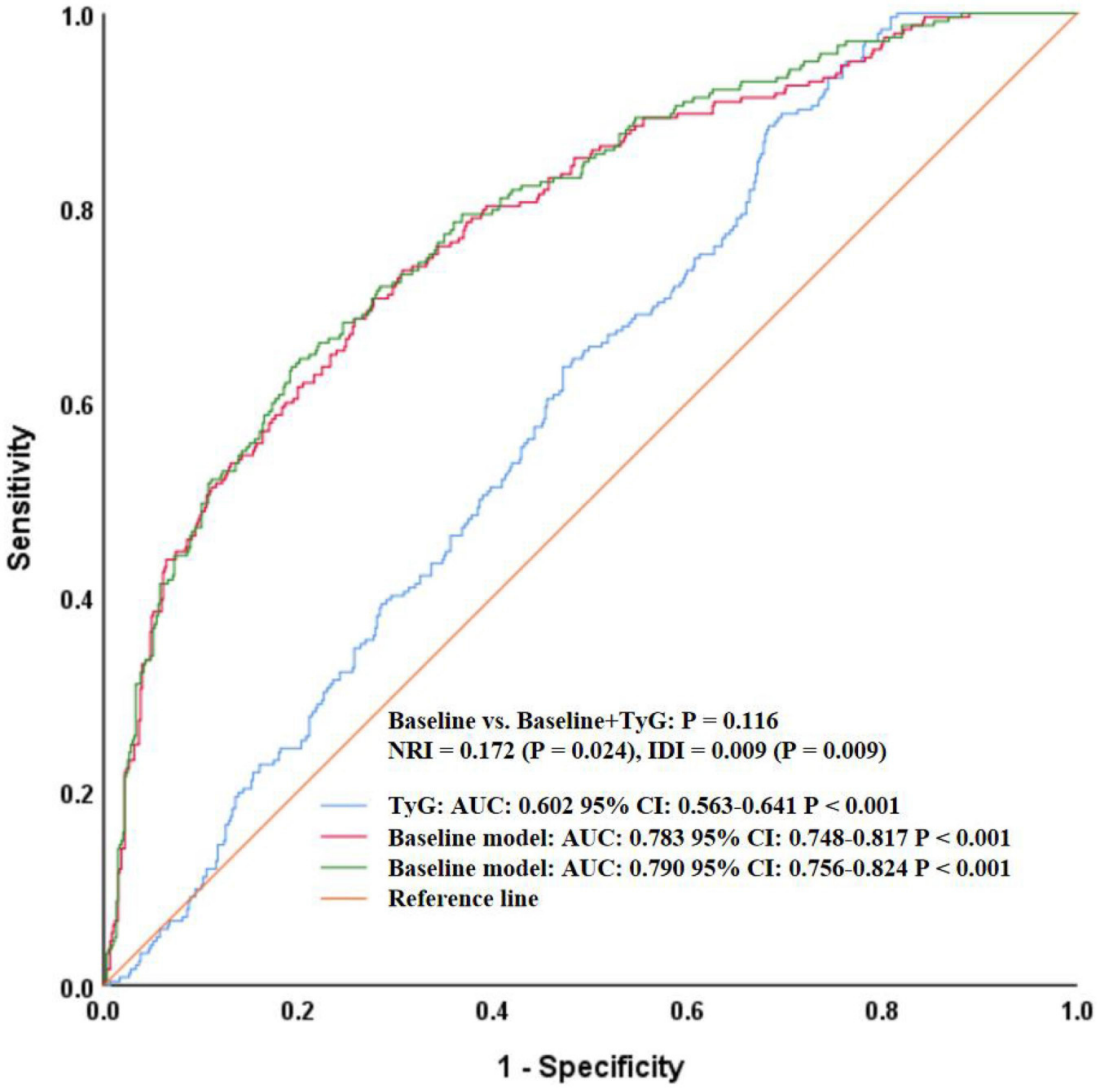
ROC curves comparing the baseline model and baseline + TyG model for heart failure prediction. ROC, receiver operating characteristic; TyG, triglyceride-glucose index; AUC, area under the curve; CI, confidence interval; NRI, net reclassification improvement; IDI, integrated discrimination improvement.

To further assess whether TyG added incremental predictive value beyond traditional variables, we constructed a baseline model including meaningful conventional variables (e.g., age, smoking, hypertension, heart rate, WBC, hemoglobin, HbA1c, HDL-C, uric acid, BUN, creatinine, AST, albumin, fibrinogen, sodium, potassium, and calcium) and then compared its performance with the model that additionally incorporated TyG (baseline + TyG). As shown in Figure 2, the AUC of the baseline model was 0.783 (95% CI: 0.748–0.817, *P* < 0.001), while that of the baseline + TyG model was 0.790 (95% CI: 0.756–0.824, *P* < 0.001). Although there was a numerical increase, DeLong's test showed the difference was not statistically significant (*P* = 0.116). However, reclassification analysis revealed that the continuous NRI was 0.172 (95% CI: 0.023–0.320, *P* = 0.024), and the IDI was 0.009 (95% CI: 0.002–0.016, *P* = 0.009), indicating that the addition of TyG significantly improved the reclassification and discrimination ability of the model.

## 4 Discussion

This study, based on a cross-sectional analysis of 848 hospitalized patients with T2DM, explored the relationship between the TyG index and HF. The findings revealed a significant association between elevated TyG levels and increased HF risk, which remained robust even after adjusting for multiple confounders including age, smoking, hypertension, heart rate, metabolic indicators, inflammatory markers, liver and renal function, and electrolytes. Subgroup analyses further demonstrated that this association was particularly pronounced among elderly patients, males, non-smokers, those with a history of hypertension, and individuals with a BMI below 24 kg/m^2^. Moreover, Although the TyG index alone showed only modest predictive ability for HF, adding it to a baseline model including traditional risk factors (e.g., creatinine) slightly improved the model's AUC, though not significantly. Notably, reclassification analysis demonstrated significant improvements in continuous NRI and IDI, suggesting that TyG may enhance risk stratification when combined with conventional variables. Overall, the study supports the clinical potential of the TyG index as a metabolic risk marker, although its discriminatory ability should be interpreted with caution, suggesting it may serve as a practical tool for identifying HF risk, particularly in high-risk T2DM populations.

In recent years, research on the association between the TyG index and HF has grown steadily, particularly within the context of metabolic disorders. For instance, Xu et al., using data from the prospective Kailuan cohort involving 138,620 participants and a median follow-up of 8.78 years, found a positive correlation between the TyG index and HF risk (19). Participants in the highest TyG quartile had a 24% higher risk of HF compared to those in the lowest quartile, and dose-response analysis revealed a significant J-shaped relationship, suggesting that the TyG index may serve as a practical surrogate marker of IR with potential clinical value for HF risk stratification. Similarly, Huang et al., in a study based on the ARIC community cohort including 12,374 participants without prior HF or coronary artery disease, observed that both baseline and long-term TyG levels were significantly associated with HF incidence over a median 22.5-year follow-up (20). An increase of one standard deviation in baseline TyG was linked to a 15% rise in HF risk, with those in the highest quartile facing a 25% increased risk. Elevated TyG levels were also associated with left ventricular structural remodeling and functional decline, indicating that TyG may not only predict HF development but also reflect subclinical cardiac impairment, thereby holding clinical value for early assessment and intervention. Zhang et al., analyzing NHANES 2007–2018 data from 13,825 participants with cardiovascular or cerebrovascular conditions, found that TyG levels were significantly higher in HF patients than in those without HF (21). Multivariate regression revealed a positive, linear dose-response relationship between TyG and HF risk, suggesting the index's potential role in risk prediction and as a therapeutic target. Similarly, Li et al. conducted a combined analysis using the Kailuan prospective cohort (*n* = 95,996) and a retrospective cohort from Hong Kong (*n* = 19,345), identifying 4,435 new HF cases (22). Participants in the highest TyG quartile had significantly greater HF risk than those in the lowest quartile. Further Mendelian randomization analysis (47,309 cases and 930,014 controls) supported a causal link between TyG and HF risk (OR = 1.27, *P* < 0.001), suggesting that TyG may not only be a predictor but also a potential mechanistic factor in HF development. Additionally, Khalaji et al. conducted a systematic review and meta-analysis of 30 studies involving 772,809 participants, concluding that elevated TyG levels were significantly associated with increased HF risk (14). The highest TyG group had a 21% higher risk of HF compared to the lowest, and each unit increase in TyG corresponded to a 17% rise in HF risk. Wang et al., in a cross-sectional study of 180 T2DM patients without prior cardiovascular disease, found that TyG was independently associated with the risk of heart failure with preserved ejection fraction (HFpEF), with a predictive AUC of 0.706, outperforming other metabolic indicators (23). This highlights the potential utility of TyG in identifying asymptomatic high-risk individuals with HFpEF for early intervention and management. Shi et al., analyzing NHANES 1999–2018 data from 13,644 individuals with diabetes or glucose regulation disorders, discovered a U-shaped association between TyG and congestive heart failure (CHF) (24). When TyG was below 8.60, higher levels were associated with reduced CHF risk, whereas above 8.60, each unit increase was linked to a 28% rise in CHF prevalence, suggesting a non-linear relationship. Furthermore, Zheng et al., using the Kailuan cohort of 56,149 participants who completed three rounds of health exams, applied latent class trajectory modeling to categorize TyG patterns from 2006 to 2010 (25). They found that individuals with persistently moderate to high or increasing TyG levels had significantly higher HF risk compared to those with consistently low TyG, indicating that long-term TyG trajectories may be valuable for dynamic HF risk assessment and reinforcing the importance of sustained glucose and lipid control in HF prevention. The findings of the present study were largely consistent with the aforementioned research but also exhibited several unique features and innovations. First, our study focused specifically on hospitalized patients with T2DM, a population with inherently higher baseline HF risk, thus offering greater clinical relevance than general population studies. Second, we employed a three-stage multivariate regression model to rigorously control for confounders, enhancing the robustness of the findings. Third, we conducted systematic subgroup analyses, uncovering potential heterogeneity in the TyG-HF association. These subgroup differences may be biologically plausible: for example, stronger associations in older adults and hypertensive patients align with the greater vulnerability of these groups to metabolic stress, endothelial dysfunction, and inflammation—mechanisms closely tied to elevated TyG. Conversely, the weaker or inverse associations seen in certain small subgroups (e.g., non-hypertensive individuals) may reflect underlying metabolic variation or simply limited statistical power. In addition, we extended the analysis beyond the TyG index alone by comparing a baseline model with and without TyG. Although the increase in AUC was not statistically significant, continuous NRI and IDI analyses demonstrated that TyG contributed meaningful incremental predictive value. This suggests that TyG may improve model-based risk classification for HF, especially when used alongside traditional variables such as creatinine.

From a mechanistic perspective, the association between the TyG index and HF remains inconclusive, yet several existing studies provide a foundation for plausible hypotheses. First, as an indirect marker of IR, an elevated TyG index may reflect impaired myocardial glucose uptake and utilization, which may disproportionately affect older individuals and males as observed in the subgroup analyses (26). Second, high TyG levels are commonly associated with chronic low-grade inflammation and oxidative stress, both of which contribute to cardiomyocyte apoptosis and fibrosis, promoting ventricular remodeling and accelerating HF progression (27–31). This mechanism could explain the stronger associations seen in hypertensive patients, in whom inflammatory burden and oxidative stress tend to be amplified. Third, TyG elevation often occurs in the context of dysregulated lipid metabolism, which may result in the accumulation of free fatty acids within myocardial tissue, inducing lipotoxic damage and impairing both the electrophysiological stability and structural integrity of the heart (32–35). Such pathways may be more relevant in individuals with low BMI, consistent with our subgroup results showing particularly strong associations in this population. Although these proposed mechanisms require further validation through experimental research, they offer a theoretical basis for future exploration.

Although this study provides relatively detailed preliminary evidence, several limitations should be acknowledged and interpreted with caution. First, due to the cross-sectional design, this study cannot establish a temporal sequence or causal relationship between the TyG index and HF, and thus the observed associations should be interpreted as correlations rather than causal inferences. Second, all data were derived from a single center, and the sample reflects specific regional and clinical settings, which may limit the generalizability of the findings, particularly because hospitalized patients from a single institution may not fully represent broader T2DM populations, highlighting the need for multicenter validation in more diverse populations. Additionally, the inclusion criterion of age ≥ 50 years—although clinically relevant given the higher HF susceptibility in older adults—may restrict the applicability of the findings to younger T2DM patients. Third, the study did not perform adequate stratified analyses for treatment factors that may affect HF risk, such as the use of novel glucose-lowering agents, which could introduce potential residual confounding. Fourth, the TyG index was measured at a single time point, and its dynamic changes and their association with HF progression were not explored, preventing assessment of longitudinal metabolic patterns that may be clinically informative. Fifth, HF was not subclassified into heart failure with reduced ejection fraction (HFrEF) and HFpEF, despite potential differences in metabolic characteristics between these subtypes that warrant further investigation in future studies, thereby limiting the granularity of phenotype-specific interpretation. The absence of subtype differentiation further constrains the ability to understand how TyG may relate to distinct HF phenotypes. Finally, some subgroup analyses involved relatively small sample sizes; thus, observed differences—especially inverse associations—should be interpreted carefully to avoid overgeneralization.

## 5 Conclusion

In conclusion, the TyG index was found to be consistently and independently associated with HF risk in hospitalized patients with T2DM; however, these findings should be interpreted as indicative of correlation rather than definitive prediction, given the cross-sectional nature of the study. While its simplicity, low cost, and ease of acquisition suggest that the TyG index may have potential utility in HF risk assessment, its clinical applicability should be considered preliminary rather than conclusive. Notably, although TyG alone demonstrated modest discriminatory ability, it provided significant incremental value in improving risk reclassification and discrimination when added to a baseline model of conventional predictors.

To strengthen the evidence base, future research should include large-scale, multicenter, longitudinal cohort studies to verify the stability and predictive performance of the TyG index, as well as mechanistic investigations to better elucidate its role in HF development. These efforts will help provide more robust tools for early identification and individualized prevention in high-risk populations.

## Data Availability

The original contributions presented in the study are included in the article/Supplementary Material, further inquiries can be directed to the corresponding author.
